# The Earthworm *Eisenia fetida* Can Help Desalinate a Coastal Saline Soil in Tianjin, North China

**DOI:** 10.1371/journal.pone.0144709

**Published:** 2015-12-23

**Authors:** Tao Zhang, Suyan Li, Xiangyang Sun, Yang Zhang, Xiaoqiang Gong, Ying Fu, Liming Jia

**Affiliations:** 1 College of Forestry, Beijing Forestry University, Beijing, China; 2 Shandong ShengWei Gardening Technology Co., Ltd., Weifang, Shandong, China; 3 Tianjin JiaLiMing Biological Technology Co., Ltd., Tianjin, China; NERC Centre for Ecology & Hydrology, UNITED KINGDOM

## Abstract

A laboratory microcosm experiment was conducted to determine whether the earthworm *Eisenia fetida* could survive in a saline soil from a field site in North China, and an experiment using response surface methodology was conducted at that field site to quantify the effects of *E*. *fetida* and green waste compost (GWC) on the salt content of the soil. The microcosm results showed that *E*. *fetida* survived in GWC-amended saline soil and increased the contents of humic acid, available N, and available P in the GWC-amended soil. The data from the field experiment were described by the following second-order model:$\hat{y}\text{ =-1.76+0}{\text{.091x}}_{\text{1}}\text{+0}{\text{.48x}}_{\text{2}}\text{-0}{\text{.00083x}}_{\text{1}}{\text{x}}_{\text{2}}\text{-0}{\text{.00078x}}_{\text{1}}^{\text{2}}\text{-0}{\text{.022x}}_{\text{2}}^{\text{2}}$, where *y* is the decrease in soil salinity (g of salt per kg of dry soil) relative to the untreated control, *x*_1_ is the number of *E*. *fetida* added per m^2^, and *x*_2_ is the quantity of GWC added in kg per m^2^. The model predicted that the total salt content of the saline soil would decrease by > 2 g kg^-1^ (*p*<0.05) when 29–90 individuals m^-2^ of *E*. *fetida* and 6.1–15.0 kg m^-2^ of GWC were applied. We conclude that the use of *E*. *fetida* for soil desalination is promising and warrants additional investigation.

## Introduction

Coastal soils in North China are threatened by salinization because of seawater encroachment and irrigation [1]. To date, over 1.3×10^5^ km^2^ of coastal lands in China have been salt-affected [2]. One of the potential solutions to this problem is bioremediation. Bioremediation has been found to be an effective and economical way to ameliorate saline soils. For example, afforestation with multiple tree species helped restore degraded sodic land [3]. Afforestation also affected other soil physico-chemical properties and soil microbial as well as enzymatic activities, making it a “relatively good option” for sustainable rehabilitation of sodic lands [4]. Likewise, perennial herbs with deep, well-developed root systems enhance soil desalinization by improving soil physical properties [5]. Although bioremediation has been widely used for salt-affected soils, most research and applications have involved planting salt-tolerant plants, and few studies have considered the use of earthworms or other soil fauna.

Earthworms are large (>2 mm wide), terrestrial, or mud-dwelling segmented invertebrates that enhance organic matter decomposition, nutrient cycling, water infiltration, and many other soil processes and properties [6]. Because earthworms are considered to be critical for the restoration of degraded ecosystems, Tao et al. (2010) suggested that earthworms could be used for saline soil amelioration [7]. Wu et al. (2001) reported that earthworms in North China could survive in soils with salinity levels as high as 11.47 g kg^-1^ [8].

In the current study, we investigated whether the earthworm *Eisenia fetida* could be used for saline soil amelioration. We selected this species because it is endemic to China; it occurs in 15 provinces including Tianjin [9]. We did not use a non-native species so as to minimize the potential negative effects of an invasive species [10]. We also selected *E*. *fetida* because it is widely cultured in China [11], making it practical for further amelioration of large areas with saline soil.

Because salinity limits the growth of many plants, plant litter, which is the major source of food for earthworms, is likely to be inadequate in areas with saline soils. Providing sufficient food for earthworms in saline soil therefore requires addition of a food supply. Green waste such as grass or flower cuttings and hedge trimmings is a suitable food for earthworms. After it is composted, semi-decomposed plant litter can be easily up taken by earthworms. In addition to supplying food for earthworms, the use of green waste helps in its disposal; green waste is generated in very large quantities in urban areas, and its disposal can be a problem.

The research reported here included a laboratory experiment and a field experiment. In laboratory microcosms, we determined whether *E*. *fetida* could survive in the coastal saline soil from Tianjin, North China, and whether its survival depended on green waste compost (GWC) amendments; we also assessed the effect of *E*. *fetida* and GWC on various soil properties. In the field, we determined how soil salinity was affected by additions of *E*. *fetida* and GWC.

## Materials and Methods

### Ethics statement

All necessary permits were obtained for the described laboratory and field experiments. The laboratory experiment was approved by the Beijing Forestry University, Beijing, China; and the field experiment was approved on this site by the Dagang Agriculture and Forestry Administration Bureau, Tianjin, China.

### Field site, GWC, and *E*. *fetida*

The field site was located in the Coastal Salt-Tolerant Plant Science and Technology Park (38°46′ N, 117°13′ E, 1.3 m a.s.l.), Dagang, Tianjin, North China. This area has a mean annual temperature of 14°C, and the mean monthly temperature ranges from -2°C in January to 28°C in July. The mean annual precipitation and evaporation are 593.6 mm and 1979.4 mm, respectively; rain that falls from June to September accounts for 84% of the annual total precipitation [12–13]. The field site had been planted with black locust trees (*Robinia pseudoacacia Linn*.) at a density of 1,111 trees per ha (based on a spacing of 3 m×3 m) 3 years before the current research has begun.

The annual peak period of soil salinization in the experimental area occurs from March to May, and the soluble salt mainly accumulates in the topsoil (0–20 cm depth) [14–15]. To ensure that the earthworms in the laboratory experiment would experience the highest levels of salinity that occurs during the year, we collected soil in March 2012 at 0–20 cm depth. Similarly, we began the field study at this field site in April 2013. No evidence of earthworms or earthworm activities was found in the soil that was collected from the site or in the soil at the site before the field experiment began. For the laboratory experiment, roots and leaf litter were removed from the soil by passing the soil through a 2-mm sieve. The physico-chemical properties of the soil were determined (Table 1).

**Table 1:** Physico-chemical properties of the saline soil from the field site.

Property	Value	
	Laboratory experiment (March 2012)	Field experiment (April 2013)
pH ^a^	7.50 (±0.05)	7.75 (±0.08)
Total salt ^a^ (g kg^-1^)	8.35 (±0.21)	8.11 (±0.35)
Clay (%)	9.5 (±0.8)	9.6 (±0.9)
Silt (%)	67.1 (±2.6)	68.0 (±2.2)
Sand (%)	23.4 (±1.7)	22.4 (±1.3)
Organic carbon (g kg^-1^)	5.74 (±0.07)	4.77 (±0.09)
Humic acid carbon (g kg^-1^)	1.90 (±0.16)	1.38 (±0.11)
Available N (mg kg^-1^)	76.91 (±0.10)	54.33 (±1.14)
Available P (mg kg^-1^)	20.84 (±2.21)	15.38 (±0.43)
Available K (mg kg^-1^)	387 (±3)	243 (±12)
Bulk density (g cm^-3^)	1.359 (±0.001)	1.366 (±0.002)

Values are means of three samples, and standard errors are in parentheses.

^a^ Determined for a slurry consisting of 1:5 (W/V) soil:distilled water.

GWC was prepared before the laboratory experiment. Green waste was obtained as part of municipal curbside collection. It was cut into small pieces (about 1-cm particle size) and subjected to a two-stage fermentation. In the first stage, the moisture content of the raw material was adjusted to 60% (W/W) and the C/N ratio was adjusted to between 25 and 30 to optimize microbial activity [16]; as recommended by Zhang et al. (2013), 0.5% brown sugar and 6% calcium superphosphate were added to improve the quality of the GWC obtained at the end of the second stage [17]. The physico-chemical properties of the GWC were determined (Table 2).

**Table 2:** Physico-chemical properties of GWC applied to saline soil.

Property	Value
pH ^a^	7.51 (±0.01)
Total salt ^a^ (g kg^-1^)	21.57 (±0.25)
Organic carbon (g kg^-1^)	375.72 (±5.99)
Humic acid carbon (g kg^-1^)	156.36 (±2.20)
Available N (mg kg^-1^)	1030.61 (±1.05)
Available P (mg kg^-1^)	242.59 (±7.18)
Available K (mg kg^-1^)	6037 (±38)

Values are the means of three samples, and standard errors are in parentheses.

^a^ Determined for a suspension consisting of 1:5 (W/V) compost:distilled water.

**Fig 1:**
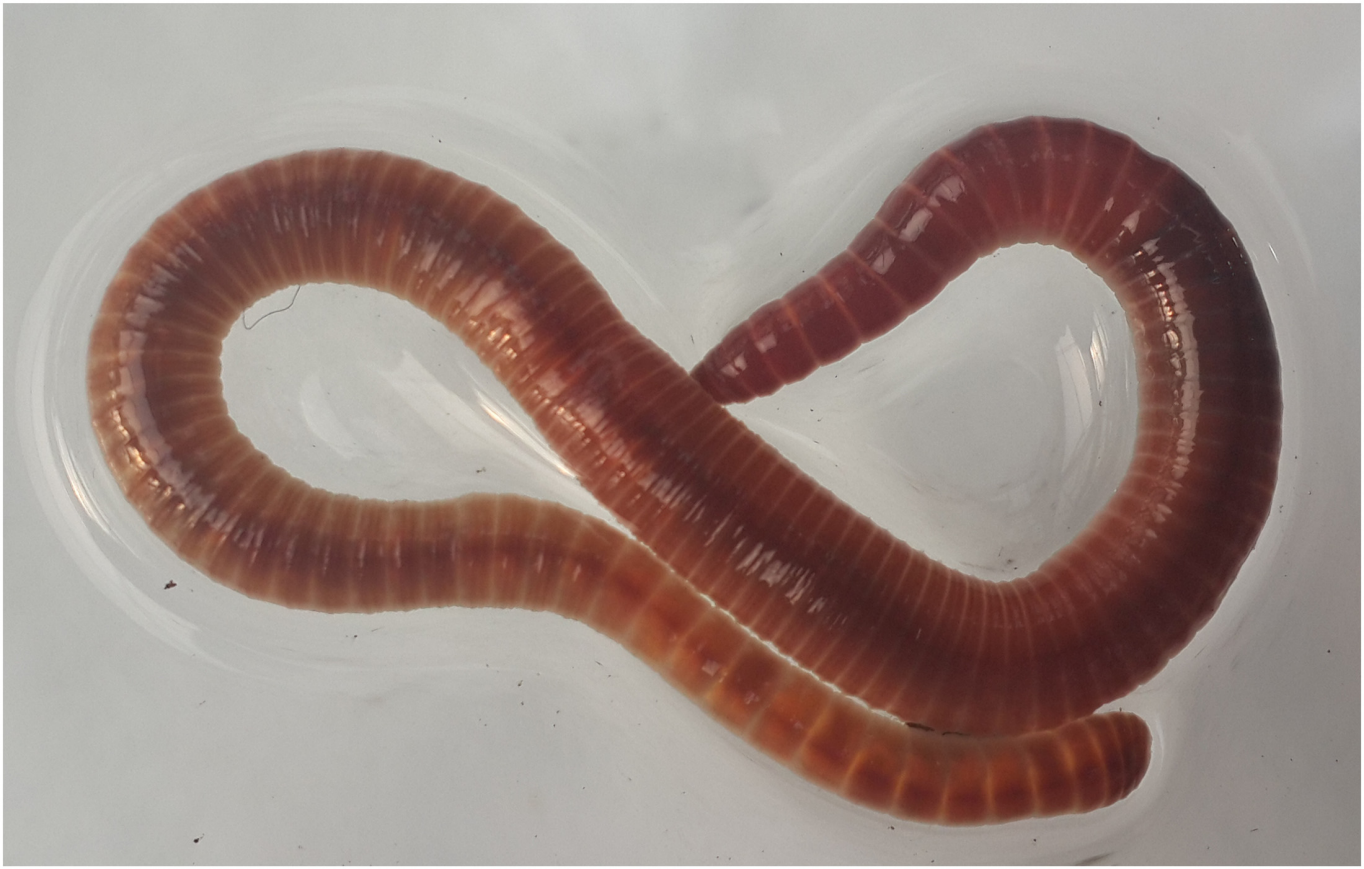
A photo of adult *Eisenia fetida* used in the study.

Adults of *E*. *fetida* (Fig 1) were collected at an earthworm hatchery in Tianjin, and were allowed to adapt to the soil conditions for 10 days by being kept in plastic jars containing the saline soil that was covered with a 1-cm layer of GWC [18]. The moisture content of the soil and GWC in the jars was 25% (±1%, W/W) [19], and the jars were kept at 20°C (±1°C) in darkness [20]. Before they were used in the laboratory experiment, the earthworms were removed from the jars and washed with distilled water to remove soil and GWC [21].

### Laboratory experiment

The laboratory experiment had a factorial design with three levels of each of two factors (number of *E*. *fetida* added and quantity of GWC added). Each microcosm (12×10×15 cm, length×width×height) was filled with 2,500 g (dry weight) of saline soil containing 0, 60, or 120 g (dry weight) of GWC. Before they were added to the microcosms, the soil and GWC were mixed and moistened to 25% (±1%, W/W) by spraying with distilled water. During the experiment, no water was added. After the microcosms were filled with soil and GWC, 0, 10, or 20 adults of *E*. *fetida* (average weight 0.47±0.003 g) were added to each microcosm. The number of earthworms added in the laboratory experiment was based on a previous laboratory study [22]. The microcosms were then sealed with nylon gauze to prevent earthworm escape. The factorial treatments and their codes are listed in Table 3. Because each of the nine treatments had three replications, the experiment included 27 microcosms. After the microcosms were sealed, they were placed randomly in a climate chamber at 20°C (±1°C) with a relative humidity of 75% (±1%). The climate chamber used in our study had a transparent glazed door (RXZ-436, China) that could get natural light, and no additional light was given during the experiment.

**Table 3:** Levels of factors used for factorial design for the laboratory experiment.

Label	Factor	Level		
*Ef*	Number of *E*. *fetida* added per microcosm	0	10	20
GWC	Quantity of GWC added (g, dry weight) per microcosm	0	60	120

GWC: green waste compost.

The laboratory experiment was conducted for 100 days because the life cycle of *E*. *fetida* requires at least 60 days under suitable situations [23]. At the end of laboratory experiment, the earthworms (including earthworm cocoons) were removed from the soil, and all of the soil was stored for analysis. The earthworms were cleaned of soil particles and counted; each cocoon was counted as 3 earthworms [24]. The daily multiplication rate of *E*. *fetida* was calculated as previously described [25]:

$$\text{daily multiplication rate = }\frac{\text{final number - initial number}}{\text{initial number × days of experiment}}$$

### Field experiment

The field experiment had a central composite design with five levels of each of two factors (number of *E*. *fetida* added and quantity of GWC added); the design consisted 14 treatments, and the levels of factors are listed in Table 4. The number of *E*. *fetida* added in the field experiment was based on Zeng (2010), who recommended the addition of 20 kg of earthworms per 667 m^2^ of forest land (equivalent to about 60 *E*. *fetida* m^-2^) [26]. GWC was added at the same rates as in the laboratory experiment. The field site was divided into 3 blocks (60×45 m), each block was again divided into 15 plots (12×15 m), hence the experiment included 45 (3×15) unit plots; each plot contained 20 trees, and the distance between adjacent blocks and plots was 3 m. The 14 treatments, along with the control group, were randomly assigned to the plots within each block and hence there were three replications of the experiment. According to Zeng et al. (2010), earthworm activity significantly improved the top 20 cm of forest soil [27], and thus the GWC was applied and mixed with the topsoil (0–20 cm depth, 1 m×1 m area around each tree) before *E*. *fetida* adults were introduced into the saline soil. For the control group, the topsoil (0–20 cm depth) around each tree was mixed but neither GWC nor earthworms were added. The field experiment was conducted for 230 days without additional artificial disturbance.

**Table 4:** Levels of factors used for central composite design for the field experiment.

Label	Factor	Level				
		-1.320 (-*γ*)	-1	0	1	1.320 (*γ*)
*x*_1_	Number of *E*. *fetida* added per m^2^	0	12	50	88	100
*x*_2_	Quantity of GWC added in kg per m^2^ (dry weight)	0	1.6	6.5	11.4	13.0

GWC: green waste compost.

The value *γ* = 1.320 was used to make the design orthogonal [28].

At the end of the field experiment, topsoil (0–20 cm depth) was collected from the amended zone (1 m×1 m around each tree) of three randomly selected trees in each plot. About 1 kg of soil was collected per plot. After drying, the soil samples were passed through a 1-mm sieve and stored in sealed polyethylene bags at 4°C until chemical analyses were performed. In May 2014, the field site was briefly examined to verify that *E*. *fetida* had survived the winter.

### Soil analysis for the laboratory and field experiments

Total salt content was determined for a slurry consisting of 1:5 (W/V) soil:distilled water by weighing the filtrate residue after the filtrate was completely dried at 105°C [29]. Humic acid carbon was extracted with 0.1 mol L^-1^ sodium pyrophosphate and quantified by the potassium dichromate combustion method [30]. Available N was determined by microdiffusion after alkaline hydrolysis. Available P was extracted with 0.5 mol L^-1^ NaHCO_3_ and was quantified by spectrophotometry (Shimadzu UV-120-02, Japan) at the wavelength of 700nm. Available K was quantified by flame photometry (FP6410, China) after NH_4_OAc neutral extraction [31].

### Statistical analysis

Multivariate ANOVAs were used to determine how the treatments affected *E*. *fetida* multiplication and soil properties in the laboratory experiment. When ANOVAs were significant, means were compared by an SNK test [32].

The response surface methodology (RSM) used for the field experiment was based on a central composite design (CCD) that consisted of four factorial points, four axial points, and six center points. The second-order model for this experiment was:

$$\hat{y}{\text{=b}}_{\text{0}}{\text{+b}}_{\text{1}}{\text{x}}_{\text{1}}{\text{+b}}_{\text{2}}{\text{x}}_{\text{2}}{\text{+b}}_{\text{12}}{\text{x}}_{\text{1}}{\text{x}}_{\text{2}}{\text{+b}}_{\text{11}}{\text{x}}_{\text{1}}^{\text{2}}{\text{+b}}_{\text{22}}{\text{x}}_{\text{2}}^{\text{2}}$$ (1)

where *y* denotes the decreases in total salt content of the saline soil (g of salt per kg of dry soil) relative to the untreated control, *x*_1_ denotes the number of *E*. *fetida* added per m^2^, and *x*_2_ denotes the quantity of GWC added in kg per m^2^. The parameter *b*_0_ represents a constant. The parameters *b*_1_ and *b*_2_ represent the linear effect of the factor *x*_1_ and *x*_2_, respectively. The parameters *b*_11_ and *b*_22_ represent the quadratic effect of factors *x*_1_ and *x*_2_, respectively, and *b*_12_ represents the interaction between factors *x*_1_ and *x*_2_.

The statistical model was developed by multiple regression analysis methods using the experimental data for the decreases in total salt content: the method of least squares estimation (LSE) was used to fit the model to the data; a test for lack of fit was then conducted to determine whether the quadratic function was sufficient for modeling the mean response. If there is no significant lack of fit, the fitted second-order model can be used to study the local response surface [33].

All statistical analyses were done using Excel 2010, SPSS 18, Design-Expert 8 and SigmaPlot 12.

## Results and Discussion

### Laboratory experiment

#### Daily multiplication rate of *E*. *fetida* in the laboratory study

**Fig 2:**
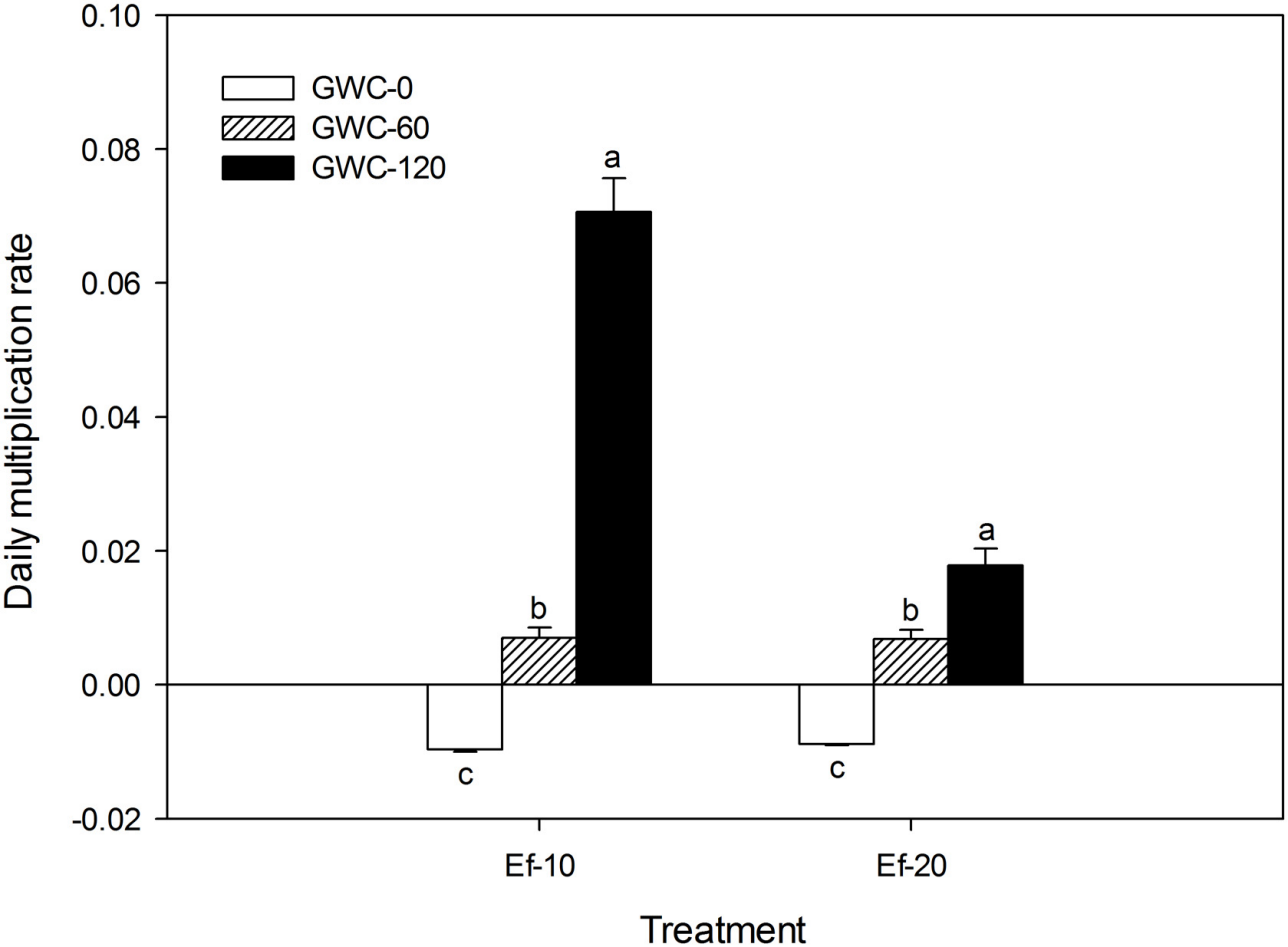
The daily multiplication rate of *E*. *fetida* as affected the quantity of GWC and number of *E*. *fetida* added in the laboratory experiment. GWC: green waste compost. Ef-*x* (*x* = 10, or 20) indicates the initial number of *E*. *fetida* added per microcosm, and GWC-*y* (*y* = 0, 60, or 120) indicates the quantity of GWC added per microcosm (g). Values are means ± SE (*n* = 3). Within each level of Ef, means followed by different letters are significantly different at *p*≤0.05 according to the SNK test.

The GWC added in this study was intended to be a food source for *E*. *fetida* and was assumed to be essential for *E*. *fetida* survival in the saline soil. That assumption was supported by the results, i.e., the daily multiplication rates of *E*. *fetida* were negative when GWC was not added and were increasingly positive as the quantity of GWC increased (Fig 2). At the highest rate of GWC addition (120 g per microcosm), the daily multiplication rate was greater when 10 rather than 20 *E*. *fetida* had been added per microcosm. Perhaps the addition of 20 rather than 10 individuals resulted in a more rapid exhaustion of the GWC or in greater intraspecific competition. Survival of *E*. *fetida* in this laboratory experiment appeared to require the addition of at least 60 g of GWC per microcosm, which is equivalent to 24 g of GWC per kg of saline soil.

#### Humic acid content of the soil in the laboratory study

**Fig 3:**
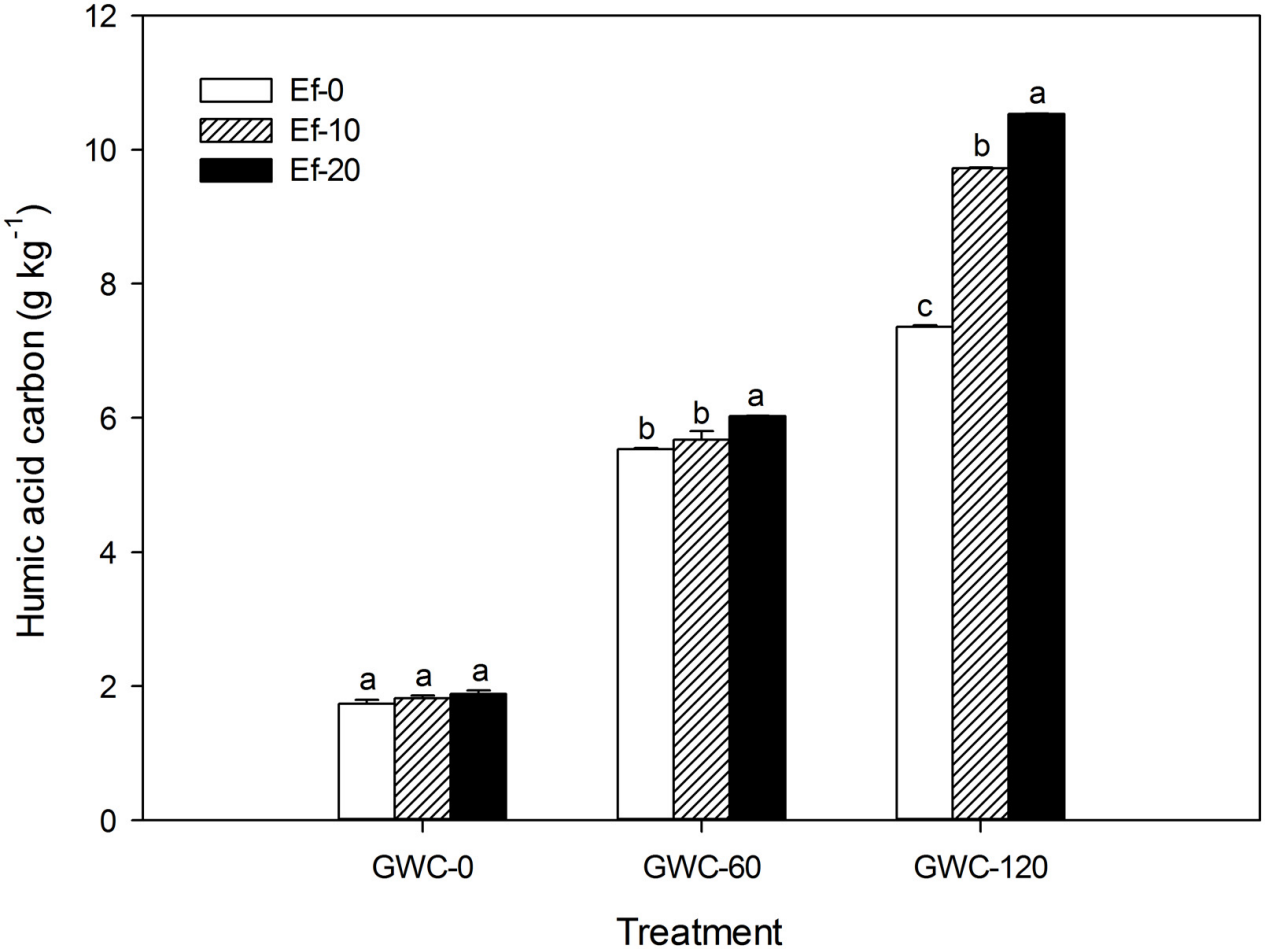
Humic acid carbon content of the soil (g kg^-1^) as affected by the number of *E*. *fetida* and the quantity of GWC added in the laboratory experiment. GWC: green waste compost. Ef-*x* (*x* = 0, 10, or 20) indicates the initial number of *E*. *fetida* added per microcosm, and GWC-*y* (*y* = 0, 60, or 120) indicates the quantity of GWC added per microcosm (g). Values are means ± SE (*n* = 3). Within each level of GWC, means followed by different letters are significantly different at *p*≤0.05 according to the SNK test.

Humic acid content clearly increased as the quantity of GWC added increased whether 0, 10, or 20 *E*. *fetida* were added to each microcosm (Fig 3). With the intermediate level of GWC addition, however, the increase was bigger with 20 than with 10 or 0 *E*. *fetida*. With the highest level of GWC addition, the increase was greatest with 20 *E*. *fetida*, intermediate with 10, and lowest with 0.

The increase in humic acid with the addition of *E*. *fetida* can be attributed to *E*. *fetida* digestion of GWC. According to Liu (2005), humic acid accounts for 7.34% of dry mass of earthworm casts [34]; this percentage may change depending on the composition of the organic material consumed by the earthworms, but there is no doubt that earthworm processing of organic materials increases the humic acid content of soils. It follows that when no GWC was added, the addition of *E*. *fetida* failed to significantly increase the humic acid content. At the same time, humic acid content can increase in the absence of earthworms because GWC contains humic acid (Table 2).

An increase in humic acid content is important for saline soil amelioration. Humic acid is a crucial component of soil humus, which has a large specific surface area (up to 2000 m^2^ g^-1^), and a cationic adsorptive capacity about 6–10 times greater than that of clay and about 24 times greater than that of silt [35]. In the neutral and calcareous soils of North China, humic acid exists in the form of calcium humate, which enhances soil structure and fertility [36]. These enhancements are essential for the leaching of salt and thus for the survival of plants.

#### Contents of available nutrients in the laboratory experiment

**Fig 4:**
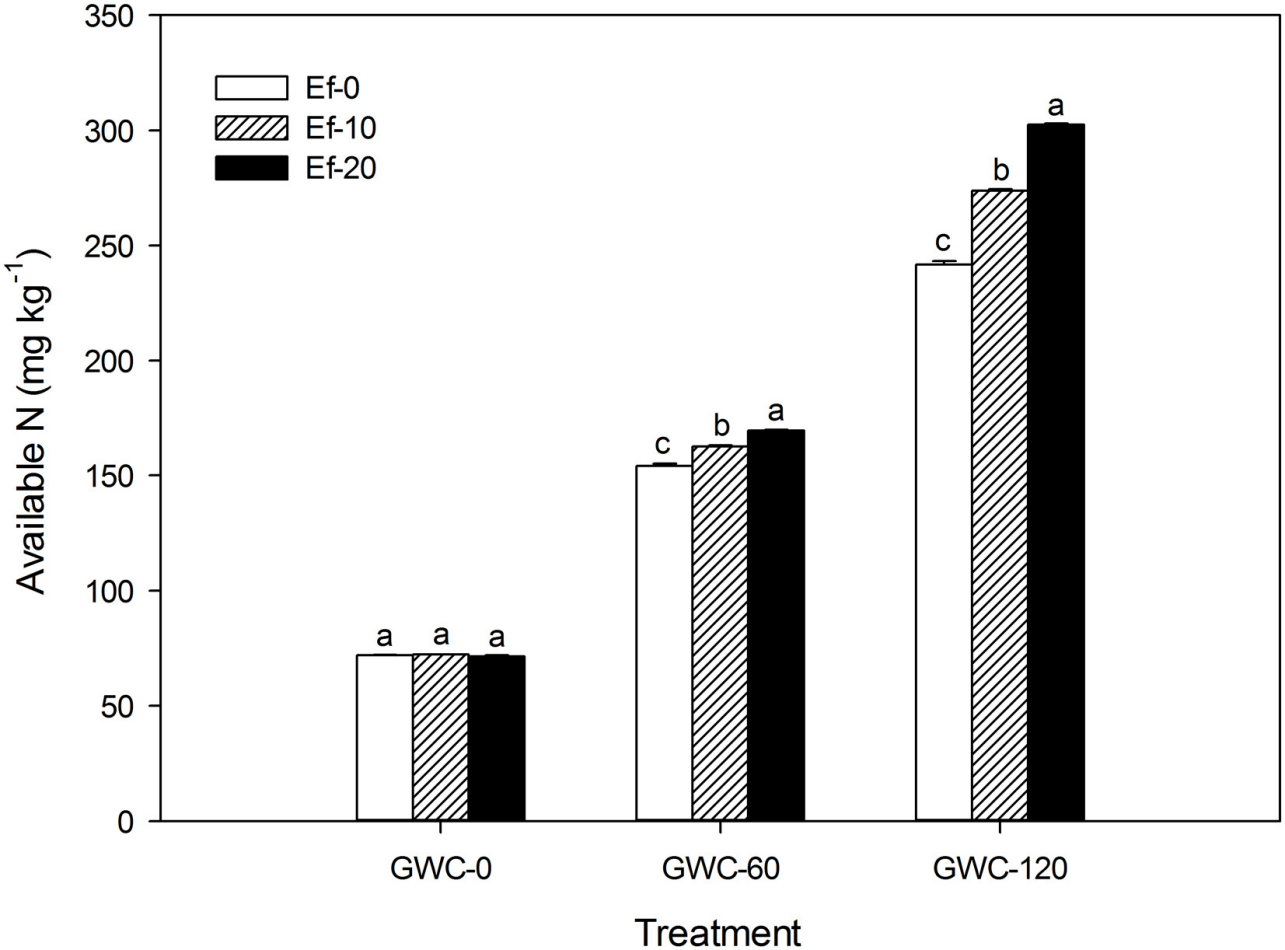
Available N content in soil (mg kg^-1^) as affected by the number of *E*. *fetida* and the quantity of GWC added in the laboratory experiment. GWC: green waste compost. Ef-*x* (*x* = 0, 10, or 20) indicates the initial number of *E*. *fetida* added per microcosm, and GWC-*y* (*y* = 0, 60, or 120) indicates the quantity of GWC added per microcosm (g). Values are means ± SE (*n* = 3). Within each level of GWC, means followed by different letters are significantly different at *p*≤0.05 according to the SNK test.

The available N content of the soil increased as the quantity of GWC added increased whether 0, 10, or 20 *E*. *fetida* were added to each microcosm (Fig 4). With the intermediate and high level of GWC addition, however, the increase was greatest with 20 *E*. *fetida*, intermediate with 10, and lowest with 0. When no GWC was added, available N content was not affected by *E*. *fetida* addition.

As with humic acid content, the increase in available N caused by *E*. *fetida* can be attributed to the digestion of GWC by *E*. *fetida*. Because the raw materials of the GWC were mainly branches and leaves collected from woody plants, the GWC probably contained substantial quantities of cellulose, hemicellulose, lignin, and organic forms of N that resist decomposition [37]. According to Cortez et al. (2000), earthworms enhance the release of N from organic matter [38]. In addition, Zhang et al. (2000) reported that earthworms enhance cellulolytic activity by increasing microbial activity [39].

**Fig 5:**
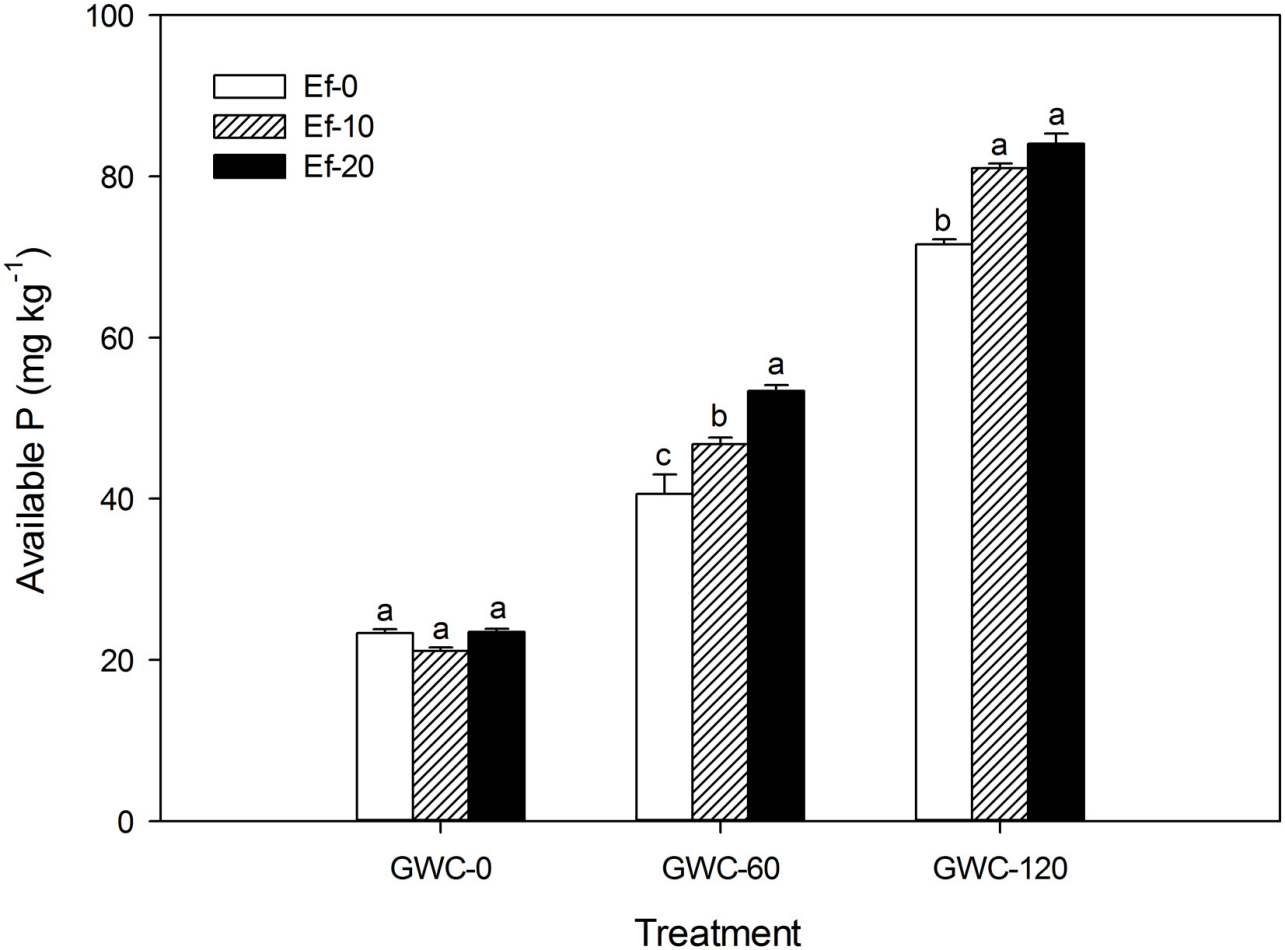
Available P content in soil (mg kg^-1^) as affected by the number of *E*. *fetida* and the quantity of GWC added in the laboratory experiment. GWC: green waste compost. Ef-*x* (*x* = 0, 10, or 20) indicates the initial number of *E*. *fetida* added per microcosm, and GWC-*y* (*y* = 0, 60, or 120) indicates the quantity of GWC added per microcosm (g). Values are means ± SE (*n* = 3). Within each level of GWC, means followed by different letters are significantly different at *p*≤0.05 according to the SNK test.

Like available N content, available P content increased with addition of GWC whether or not *E*. *fetida* was added (Fig 5). Available P content was further increased by addition of *E*. *fetida* when GWC was added but not when GWC was not added. The increase in P availability resulting from *E*. *fetida* addition can be explained in two ways. First, organic P, which is unavailable to plants and represents from 20 to 50% of the total P in soil, can be mineralized and made available by earthworm activities and enzymes [40]. Second, by adsorbing to clay minerals and hydroxides, humic acid could reduce the adsorption of P to these materials and thus reduce P immobilization [36]. As indicated, these increases in P availability depended on the addition of GWC to soil.

In contrast to the findings of Wu et al. (2013) [41], the addition of *E*. *fetida* did not significantly affect available K content (S1 Fig). Our findings differed from those of Wu et al. perhaps because the GWC in the current study contained a high level of available K (6037 mg kg^-1^, dry weight). In addition, Wu et al. added cow dung rather than GWC.

The increases in available N and available P caused by the addition of *E*. *fetida* are of course conducive to plant growth. Hence, we conclude that earthworms could indirectly improve the saline soil by enhancing plant growth [3–5].

#### Total salt content in the laboratory experiment

Because the microcosms used in the laboratory study were closed, leaching could not occur, and the salt content of the soil was unaffected by the presence of *E*. *fetida* (S2 Fig).

### Field experiment

#### Fitting the second-order model to the field data

Addition of GWC and *E*. *fetida* reduced the levels of salt in the soil, and the reductions relative to the untreated control (*y*) are shown in Table 5 (the total salt content in soil of the untreated control was 7.31±0.10 g kg^-1^). A second-order model was fitted by multiple regression analysis methods using the experimental data for the decreases in total salt content, which could be described as:

$$\;\hat{y}\text{ =-1.76+0}{\text{.091x}}_{\text{1}}\text{+0}{\text{.48x}}_{\text{2}}\text{-0}{\text{.00083x}}_{\text{1}}{\text{x}}_{\text{2}}\text{-0}{\text{.00078x}}_{\text{1}}^{\text{2}}\text{-0}{\text{.022x}}_{\text{2}}^{\text{2}}$$ (2)

**Table 5:** Decreases in total salt content of the soil relative to the untreated control in the field study; *γ* = 1.320.

Treatment	Code values		Actual values		*y*: Decrease in total salt relative to the untreated control
	*X*_1_	*X*_2_	*x*_1_	*x*_2_	(g kg^-1^)
T1	1	1	88	11.4	1.92 (±0.03)
T2	1	-1	88	1.6	0.92 (**±**0.02)
T3	-1	1	12	11.4	1.65 (**±**0.03)
T4	-1	-1	12	1.6	0.03 (**±**0.02)
T5	*γ*	0	100	6.5	1.18 (**±**0.04)
T6	-*γ*	0	0	6.5	0.44 (**±**0.02)
T7	0	*γ*	50	13.0	3.05 (**±**0.09)
T8	0	-*γ*	50	0	0.67 (**±**0.04)
T9	0	0	50	6.5	3.52 (**±**0.12)
T10	0	0	50	6.5	2.94 (**±**0.04)
T11	0	0	50	6.5	2.50 (**±**0.09)
T12	0	0	50	6.5	3.22 (**±**0.17)
T13	0	0	50	6.5	2.03 (**±**0.04)
T14	0	0	50	6.5	2.45 (**±**0.14)

T1-T4 were factorial points, T5-T8 were axial points, and T9-T14 were center points.

Values for *y* are means of three replications, and standard errors are in parentheses.

The results of ANOVA statistics (S1 Table) showed that the relationship between the model and the response variables was statistically significant (*p* = 0.0008), which means the model (2) could reasonably describe the data concerning the decrease in the salt content of the soil resulting from the addition of *E*. *fetida* and GWC in the field experiment. Moreover, the lack-of-fit test for the model was insignificant (*p* = 0.9439), which indicated that the model (2) was fitted adequately. The results also showed that the model (2) explained 83.62% of the variability of the response data around its mean, and predicted R-squared of 0.8153 was in reasonable agreement with the adjusted R-squared of 0.8362, which means the model could provide valid predictions for the field experiment.

#### Response surface and predictions of the second-order model of the field study

**Fig 6:**
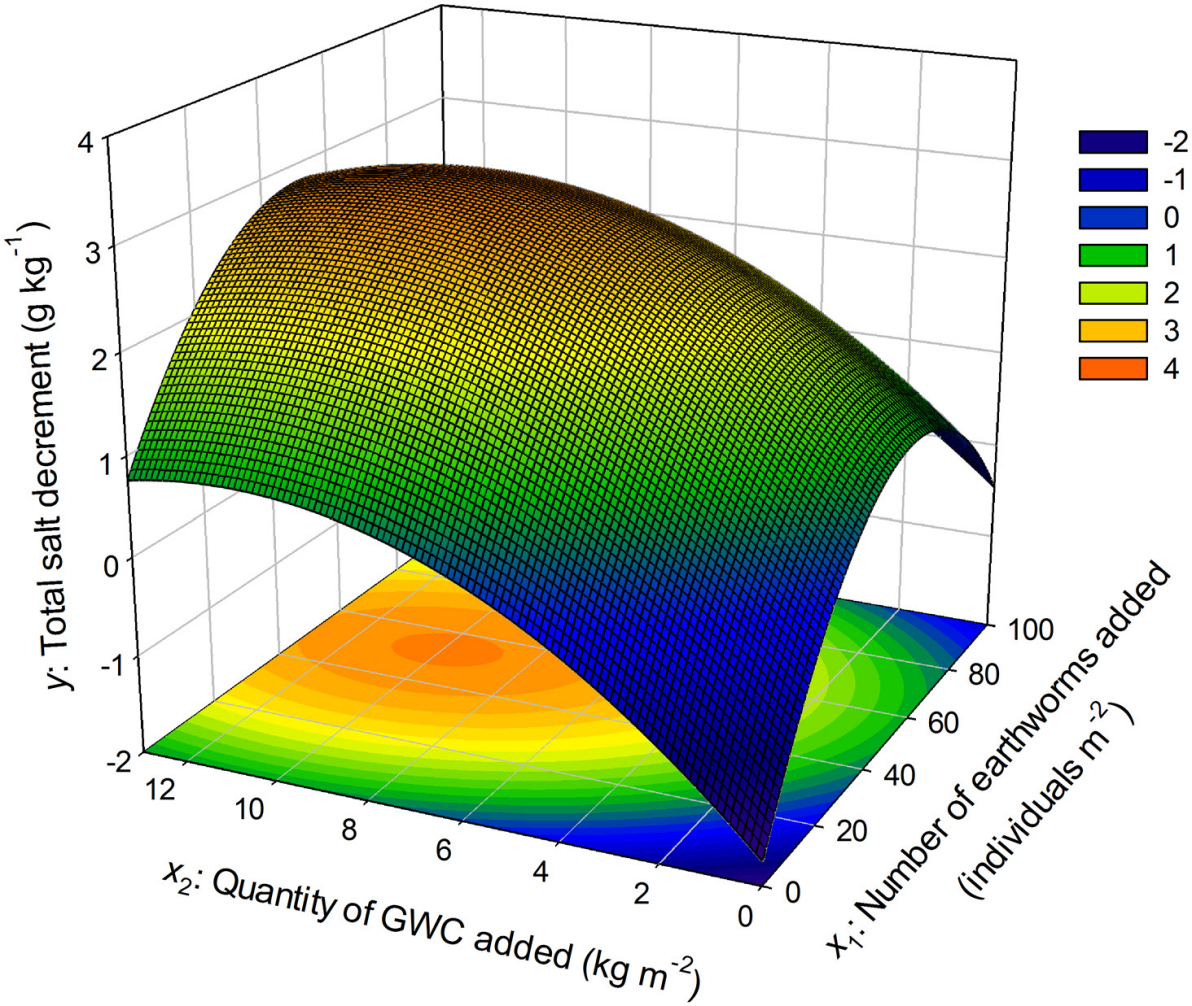
Response surface for the decrease in total salt content of the soil as affected by the number of *E*. *fetida* and quantity of GWC added in the field experiment. GWC: green waste compost. The fitted model for the response surface was: $\hat{y}\text{ =-1.76+0}{\text{.091x}}_{\text{1}}\text{+0}{\text{.48x}}_{\text{2}}\text{-0}{\text{.00083x}}_{\text{1}}{\text{x}}_{\text{2}}\text{-0}{\text{.00078x}}_{\text{1}}^{\text{2}}\text{-0}{\text{.022x}}_{\text{2}}^{\text{2}}$

The response surface based on model (2) is presented in Fig 6. The model predicted that the stationary point (the point of maximum response) was located at (53, 9.9), which means that a maximum decrease in total salt content (*y*_max_) of 3.03 g kg^-1^ occurred when 53 *E*. *fetida* m^-2^ and 9.9 kg of GWC m^-2^ were applied to the saline soil of the experimental site. The model also predicted that when 29 to 90 *E*. *fetida* m^-2^ and 6.1 to 15.0 kg of GWC m^-2^ were applied to the saline soil of the experimental site, the total salt content would be reduced by > 2 g kg^-1^ (*p*<0.05), i.e., that the soil salinity would be reduced by about 27% relative to the untreated control.

The effects of *E*. *fetida* on soil desalination under the field conditions may be explained by two general mechanisms. First, the burrows, diapause chambers, and casts generated by *E*. *fetida* would accelerate the formation of soil pores and soil aggregates [42], which in turn would enhance water infiltration and drainage [6] and thus increase salt leaching. Second, *E*. *fetida* would likely accelerate the decomposition of organic materials and thus increase available nutrient contents, which would enhance plant growth and health [43–45] and thus increase the contribution of plants to soil desalination.

#### Possibilities of long-term amelioration

Although *E*. *fetida* numbers at the end of the field experiment were not determined, burrows, fresh worm casts, and living earthworms were observed when the field site was briefly examined in May 2014. This suggested that *E*. *fetida* might be able to persist under the conditions of our field experiment. The establishment of *E*. *fetida* populations in such field sites would be important for the long-term amelioration of saline soils by earthworms because it would not be practical to replace earthworm losses every year. Repeated addition of GWC may also not be necessary if the field sites produced sufficient litter to sustain *E*. *fetida* populations. The positive feedback between earthworms and plants (earthworms enhance plant growth by mineralizing nutrients and by improving soil structure while plants enhance earthworms by providing food in the form of litter) might contribute to the long-term amelioration of saline soils.

## Conclusions

Based on the results of the laboratory experiment, we conclude that the earthworm *E*. *fetida* can survive in a typical saline soil of North China if the soil is supplemented with GWC. The laboratory results also indicated that *E*. *fetida* can significantly increase the humic acid, available N, and available P in saline soil amended with GWC.

The results of the field experiment indicated that *E*. *fetida* can contribute to soil desalination. The effect of additions of *E*. *fetida* (*x*_1_, number of individuals added per m^2^) and GWC (*x*_2_, kg added per m^2^) on the decrease in soil salinity (*y*, g of salt per kg of dry soil) relative to the untreated control was described by the following second-order model: $\hat{y}\text{ =-1.76+0}{\text{.091x}}_{\text{1}}\text{+0}{\text{.48x}}_{\text{2}}\text{-0}{\text{.00083x}}_{\text{1}}{\text{x}}_{\text{2}}\text{-0}{\text{.00078x}}_{\text{1}}^{\text{2}}\text{-0}{\text{.022x}}_{\text{2}}^{\text{2}}$. With this model, we predict that the total salt content of the saline soil in the current study would decrease by > 2 g kg^-1^ (*p*<0.05) when 29 to 90 *E*. *fetida* and 6.1 to 15.0 kg of GWC are added per m^-2^; this represents a 27% reduction in soil salinity relative to the untreated control. We also found evidence (burrows, fresh worm casts, and living earthworms) indicating that *E*. *fetida* could survive the winter at the experimental site.

Because the salt tolerance of earthworms is limited, we suggest introduction of *E*. *fetida* after other ameliorations (such as soil dressing, tile drainage, and addition of soil conditioner) are applied to soils that contain more salt than the 8.35 g kg^-1^ in the soil used in the laboratory study. Although *E*. *fetida* can tolerate higher soil salinity than other earthworm species, they do not move below 20 cm depth, and this limits their contribution to saline soil improvement. Future research should consider the use of endogeic earthworm species that move deeper into the soil than *E*. *fetida*. Endogeic species might be particularly useful if added after *E*. *fetida*.

## Supporting Information

S1 Fig Available K content in soil (mg kg^-1^) as affected by the number of *E*. *fetida* and the quantity of GWC added in the laboratory experiment. (TIF)

S2 Fig Total salt content in soil (g kg^-1^) as affected by the number of *E*. *fetida* and the quantity of GWC added in the laboratory experiment. (TIF)

S1 Table Analysis of variance concerning the effects of *E*. *fetida* and GWC on the decreases in total salt content of soil in the field experiment. (PDF)
