# Supplementary figures and images for: The Earthworm Eisenia fetida Can Help Desalinate a Coastal Saline Soil in Tianjin, North China
Source: PLoS One. 2015 Dec 23;10(12):e0144709. doi: 10.1371/journal.pone.0144709 (PMC4689387; doi:10.1371/journal.pone.0144709)

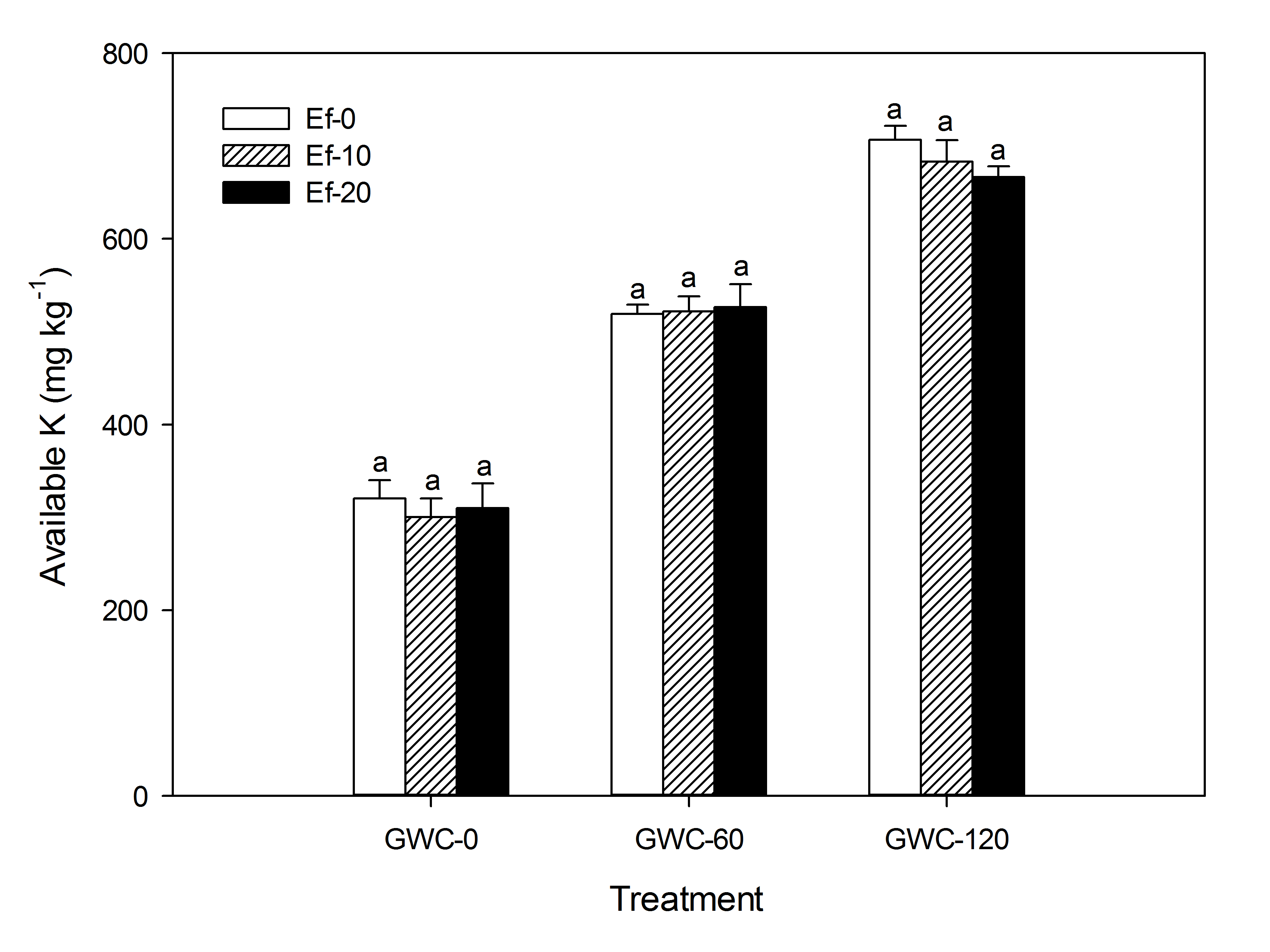

Supplement: S1 Fig — (TIF) [file pone.0144709.s001.TIF]

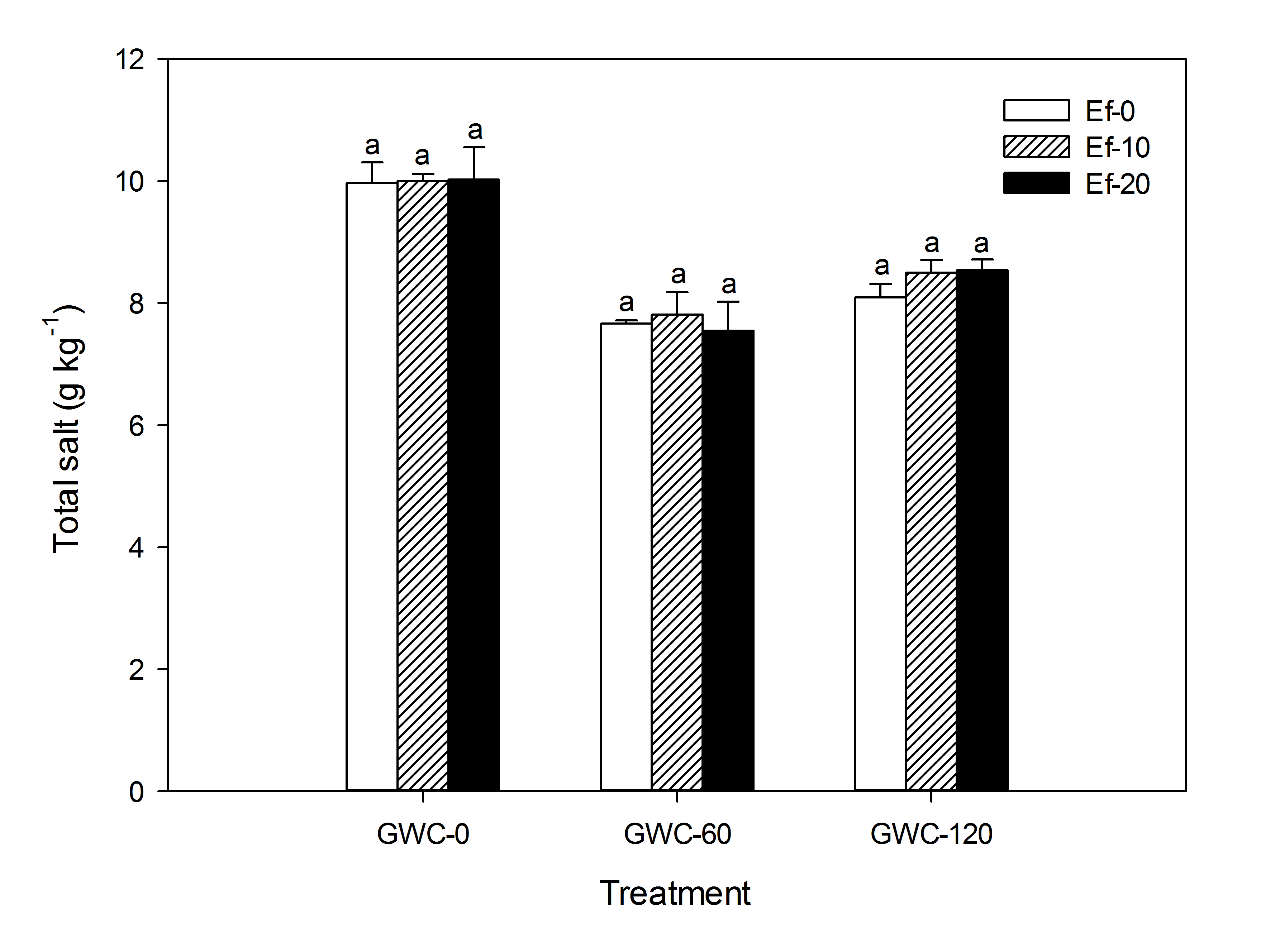

Supplement: S2 Fig — (TIF) [file pone.0144709.s002.TIF]
