# Supplementary material for: The Earthworm Eisenia fetida Can Help Desalinate a Coastal Saline Soil in Tianjin, North China
Source: PLoS One. 2015 Dec 23;10(12):e0144709. doi: 10.1371/journal.pone.0144709 (PMC4689387; doi:10.1371/journal.pone.0144709)
Supplement: S1 Table — (PDF) [file pone.0144709.s003.pdf]

S1 Table. Analysis of variance concerning the effects of *E. fetida* and GWC on the decreases in total salt content of soil in the field experiment.

**Source of variation**	**Sum of squares**	**Degrees of freedom**	**Mean square**	***F*-value**	***p*-value**
Model	14.4874	5	2.8975	14.2760	0.0008
x_1	0.6100	1	0.6100	3.0056	0.1215
x_2	4.4351	1	4.4351	21.8522	0.0016
x_1x_2	0.0961	1	0.0961	0.4735	0.5112
x_1^2	7.6881	1	7.6881	37.8799	0.0003
x_2^2	1.6580	1	1.6580	8.1690	0.0215
Residual	1.6237	8	0.2030		
Lack of fit	0.1178	3	0.0393	0.1304	0.9439
Pure error	1.5059	5	0.3012		
Cor Total	16.1110	13			

GWC: green waste compost.

$p<0.05$ indicates that the effect was significant.
